# Effect of an herbal supplement on quality of life in participants with insomnia: A randomized placebo controlled cross-over pilot trial

**DOI:** 10.1371/journal.pone.0350039

**Published:** 2026-05-20

**Authors:** Prachi Singh, Isabella Griffith, Makayla Tanksley, Robbie A. Beyl, Frank Greenway

**Affiliations:** Pennington Biomedical Research Center, Baton Rouge, Louisiana, United States of America; Johns Hopkins: Johns Hopkins University, UNITED STATES OF AMERICA

## Abstract

**Background:**

Insomnia is a common sleep disorder linked to poor quality of life. Patients with insomnia frequently rely on herbal supplements to improve sleep and quality of life; however, most supplements do not undergo rigorous testing to determine their effectiveness. The primary objective of the pilot trial was to test the effectiveness of the herbal supplement on sleep and quality of life.

**Methods:**

In a double-blind, randomized, placebo-controlled, crossover pilot trial, participants were assigned study drinks consisting of the herbal supplement containing extracts from chicory and green tea or placebo for 7 days. This was followed by a crossover to the other arm after 2 weeks of a washout period. Participants were healthy individuals of the age of 50 years or more with insomnia. Study measures included subjective assessments of sleep, daytime sleepiness, fatigue, and quality of life via questionnaires. Habitual sleep patterns and sleep architecture were objectively evaluated by a 7-day accelerometry and polysomnography.

**Results:**

Ten participants (9 women; age 59 ± 6 y; BMI 30 ± 10 kg/m^2^) with insomnia severity index of 21 ± 4 and wake after sleep onset (WASO) of 75 ± 33 min were enrolled in the study. The impact of placebo and supplement was different for measures related to daytime sleepiness and physical fatigue but not quality of life. No within and between treatment differences were observed for any objective measures of habitual sleep patterns or sleep architecture. Within group analysis showed improvement in subjective sleep quality, daytime sleepiness, and general fatigue after 7-day consumption of the placebo drink, but not after consumption of herbal supplement. There were no adverse events related to study participation.

**Conclusion:**

Compared to placebo drink, daily consumption of the herbal supplement for 7 days did not improve any aspect of quality of life or sleep in our pilot study participants with insomnia. Well controlled clinical trials are needed to confirm the claims related to supplements.

## Introduction

Sleep and quality of life are closely entwined such that sleep disorders like insomnia are closely linked to poor quality of life [1]. It is important to gain an understanding of factors associated with poor quality of life as a patient’s perception of quality of life can predict treatment and intervention outcomes [2]. Notably, gaining insights into quality of life can improve therapeutic strategies for symptom relief, care, and rehabilitation of patients, if needed. Indeed, treatment of insomnia is shown to reduce health care costs and improve quality of life [3].

Among sleep disorders, insomnia remains prominent with one third of the adult population reporting dissatisfaction with sleep and presence of insomnia symptoms [4]. Further, 5% to 15% of adults meet formal diagnostic criteria for chronic insomnia. Based on the 2020 data from the National Health Interview survey, 14.5% of adults had trouble falling asleep, and 17.8% of adults had trouble staying asleep most days in a 30-day period [5]. While the relationship between insomnia, cardiometabolic risk, and quality of life is recognized, few physicians routinely conduct full sleep histories on their patients, and the patient satisfaction for insomnia treatment remains low [6]. Clinical recommendations for treatment of insomnia include cognitive behavior therapy and prescription drugs [4]. However, cognitive behavior therapy remains underutilized, and patients are often weary of using prescription drugs due to undesirable side effects, limited effectiveness, and potential for dependency [4, 6]. Hence, individuals with insomnia frequently seek supplements to improve their sleep and quality of life.

Several commonly used over-the-counter supplements claim to improve sleep-related quality of life and are potentially cost-effective with minimum side effects. However, most supplements do not undergo rigorous and controlled clinical trials. This contributes to inconsistent effects on sleep and quality of life. Here, we conducted a double-blind, placebo-controlled, randomized, crossover pilot trial to examine the effects of a supplement containing additives believed to improve sleep and quality of life. The pilot study aimed to 1) generate preliminary data to support claims that the supplement may be effective in improving quality of life and/or sleep; and 2) establish the statistical estimates required to power a larger definitive clinical trial that is able to test if supplement a) alters quality of life; and b) changes in quality of life are correlated with changes in sleep efficiency. Our supplement contained derivatives from decaffeinated green tea and chicory which are shown to improve sleep in animal models and anecdotally in humans [7–10]. We hypothesized that the daily consumption of the supplement would improve quality of life, habitual sleep patterns and sleep architecture.

## Methods

The study received ethical approval from the Pennington Biomedical Research Center (PBRC) Institutional Review Board (Protocol ID: 2020–039-PBRC Sleep Lift) prior to study initiation. The study was conducted at PBRC located in Baton Rouge, Louisiana, USA in accordance with the 1975 Helsinki Declaration, and all participants provided written informed consent prior to any study procedures. The study was retrospectively registered at ClinicalTrials.gov [NCT05323084] within 30 days of enrollment of the first study participant. The delay in study registration was due to unavoidable administrative and logistic issues. The authors confirm that all ongoing and related trials for this drug/intervention are registered. Study recruitment started on Jan 4, 2022 and ended on September 7, 2022 (after planned enrollment of 10 participants). The first participant was enrolled on Feb 25, 2022.

### Participants

**Fig 1:**
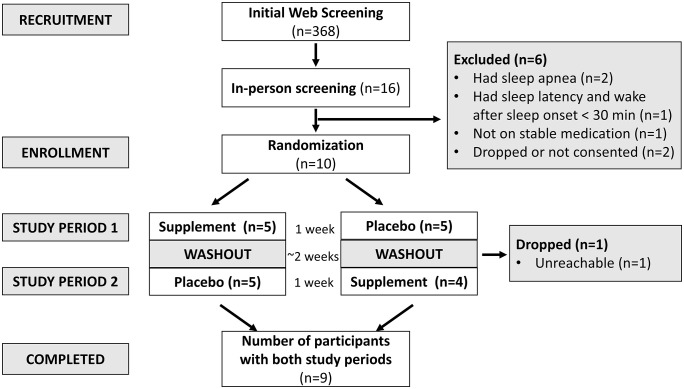
Participant flow diagram. Interested volunteers were prescreened via a web-based screener and a phone call. Potentially eligible participants had an in-person screening visit and eligibility confirmed. Participants were randomly assigned to first receive supplement or placebo drink followed by at least 2 weeks washout period and taking the other remaining drink. All available data from the enrolled participants was used for the analysis (n = 10).

The study included individuals over the age of 50 y with habitual bedtime between 9 pm and midnight and without any restriction to race, BMI, or gender. By not including participants with <50 y of age, confounding issues related to age and menopause status were minimized for this pilot study. Importantly, insomnia is prominent in older individuals, especially women. The inclusion criteria were the presence of insomnia but the absence of sleep apnea. Other exclusion criteria included diabetes, use of sedating or hypnotic medications, or any chronic medication for which the dosage changed within the last 1 month. A full list of inclusion and exclusion criteria are presented in S1 Table. Participants were recruited from the community via email lists, flyers, social media, and website advertisements. Participant flow during the clinical trial is outlined in Fig 1.

### Trial design

**Fig 2:**
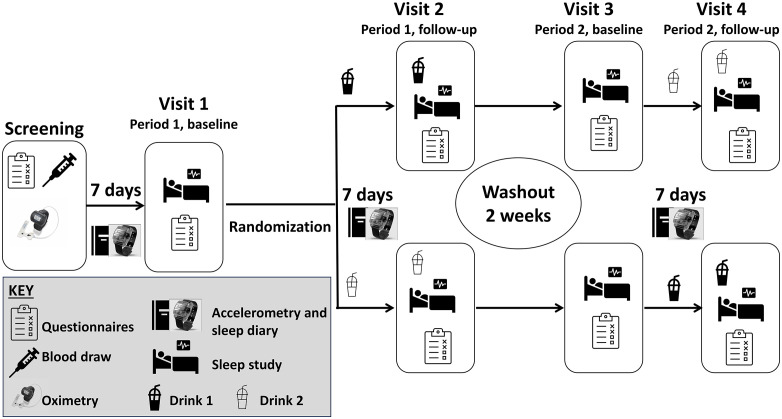
Schematic study design. Participant eligibility criteria were determined by brief medical history and physical followed by questionnaire-based assessment of insomnia, blood draw, overnight oximetry, and 7-day accelerometry accompanied with sleep diary. During the study, participants underwent an overnight sleep study followed by questionnaire-based assessment of quality of life, daytime sleepiness, fatigue, and self-perceived sleep quality. Following the baseline assessment, participants were randomized and provided study drink consisting of either placebo or supplement to take 1 - 2 h prior to intended bedtime for the next 7 days. During this period, the participant also maintained a sleep diary and wore an accelerometer. On the seventh night, participants returned for the sleep study and questionnaire-based assessment. At this time, the participant took the placebo/supplement 1 h prior to intended bedtime. After a 2-week washout period the study baseline and follow-up assessments were repeated, and the remaining study drink was provided.

We conducted a double-blind, placebo-controlled, randomized, crossover pilot clinical trial to measure the effect of a dietary herbal supplement on the quality of life and sleep in individuals with insomnia (Fig 2). Eligible participants underwent a baseline in-lab sleep study and were asked to fill out validated questionnaires to assess perceived quality of life, daytime sleepiness, sleep quality, and fatigue. Following baseline assessments, participants were randomly assigned to either take the placebo or supplement drink for the next 7 days. The intervention duration was based on anecdotal evidence which suggested an acute same day effect on sleep. The 7-day duration would test if the beneficial effects on sleep would be sustained. The participants were instructed to take the study drink 1–2 h prior to intended bedtime. In addition, participants maintained a sleep diary and wore an accelerometer on the wrist during this period. On the 7^th^ night, participants returned to PBRC for a follow-up sleep study and were given the assigned drink prior to the polysomnography. We asked for the questionnaires to be filled out again the following morning. This was followed by a 2-week washout period and then a crossover to the other arm. The washout duration was based on a prior randomized crossover clinical trial that examined the impact of cherry juice on sleep in participants with insomnia [11]. Baseline measures were reassessed for each study period except for habitual sleep patterns that were derived as 7-day averages including the entire study/intervention period. The study ended on the fourth study visit. No changes to the trial design were made after the commencement of the study.

### Screening visit

This visit provided study-related information to the participants and verified eligibility criteria. After obtaining written informed consent, blood was drawn in fasting conditions, and the participant’s body weight and height were determined. Screening questionnaires were administered to establish the presence of insomnia, daytime sleep related impairment, and absence of any chronic health conditions with unstable medications. Insomnia was determined by 1) self-report of chronic difficulties sleeping; 2) an insomnia severity index (ISI) questionnaire score of ≥10 [12]; and 3) accelerometer data of 7-days showing a minimum average score of 30 min for either sleep latency or wake after sleep onset (WASO) [4]. Chronic difficult sleeping was defined as self-report of having trouble initiating sleep, maintaining sleep, or early awakening, on average, a minimum of 3 nights per week for the duration of at least 6 months. Importantly, sleep difficulties occur despite adequate sleep opportunity and are associated with some daytime consequences such as fatigue, poor mood, social/work dysfunction, or irritation. To ensure the absence of sleep apnea, participants were objectively screened by overnight oximetry (Model 3150 WristOx2® Pulse Oximeter, Nonin), and individuals with an oxygen desaturation index of >5 events/h of sleep were not included [13]. Fasting blood draws measured glucose, lipid profiles, liver enzymes, and creatinine to determine health and ensure participant safety. Blood measure used standard clinical laboratory protocols.

### Study procedures

No changes to the pilot trial assessments were made after the commencement of the study.

#### Polysomnography (PSG).

Sleep architecture, breathing events, and limb movements were measured by an in-lab complete polysomnography including electroencephalography, electrooculography, electromyography, electrocardiography, pulse oximetry, nasal pressure transducer, oronasal thermocouple, and calibrated respiratory inductance plethysmography (Grael Compumedics, Melbourne, Australia). All participants were given an equal sleep opportunity from 10:00 pm to 6:00 am, unless participant preferred to sleep earlier or had a spontaneous early awakening (~5:00 am) and did not want to stay in bed. The study was manually scored in 30-second epochs by a registered PSG technician according to established criteria [14].

#### Accelerometer.

Habitual sleep patterns were determined using a 7-day wrist worn accelerometer (ActiGraph^TM^ wGT3X-BT). The accelerometer was worn on non-dominant hand, and a sleep diary was maintained at the same time. The data collection rate was set at 80 Hz, the data was downloaded in 60 sec epochs, and the sleep-related information was determined using the Cole-Kripke and Tudor-Locke algorithms. If needed, daily sleep periods detected via the algorithm were manually altered to align with the bedtime reported in the sleep diary [15]. Averages across the 7-day intervention duration during both study periods were assessed to examine habitual sleep patterns.

#### Questionnaires.

**Chubon life situation survey (LSS):** Chubon LSS is a 20 item Likert-type scale designed to measure perceived life quality with high scores reflecting higher perceived life quality and a score range of 20–140 [16]. The items on this questionnaire are derived from several aspects of life and are considered relevant to a broad population. In general, scores of 100 or higher reflect the perception of a very good quality of life. The survey has undergone several reliability and validity studies with a coefficient ranging from 0.74 to 0.95 (Cronbach’s alpha) in populations ranging from prison inmates, university students, and patients undergoing rehabilitation for spinal cord injury [16].

**Epworth sleepiness scale (ESS):** ESS is a validated scale used to estimate daytime sleepiness with a score range of 0–24 and scores >10 are considered as excessive daytime sleepiness [17]. The questions in this survey examine the chances of falling asleep in eight different situations such as reading, watching TV, etc. The ESS scores are known to be reliable in a test-retest sense over a period of months with Cronbach’s alpha statistics in the range of 0.88–0.74 [17]. The ESS scores are significantly associated with self-reported problem of sleepiness but have low correlation with mean sleep latency (an objective measure of daytime sleepiness).

**Pittsburgh sleep quality index (PSQI):** PSQI provides an estimate of self-reported sleep quality [18]. The overall scores range from 0 to 21 with higher scores indicating worse sleep quality. An overall score of >5 is associated with poor sleep quality. While standard PSQI questions relate to sleep during the past month, we asked the participant to answer based on the last 7 days as the study intervention is for 7 days. PSQI test-retest reliability coefficient is high (0.87) and the index has a good validity for patients with primary insomnia [19].

**Multidimensional fatigue inventory (MFI):** MFI is a 20-item scale designed to evaluate five dimensions of fatigue—general fatigue, physical fatigue, reduced motivation, reduced activity, and mental fatigue [20]. The score for each domain ranges from 4–20, with higher total scores corresponding with elevated levels of fatigue. The test-retest reliability is reported to be 0.84 (Cronbach’s alpha) in different populations including cancer patients, university students, and patients with chronic fatigue syndrome [20].

#### Randomization and blinding.

After baseline study measures were obtained, the participants were randomly assigned to first receive placebo or supplement (1:1). A block randomization method was used to determine crossover order (placebo first Vs supplement first). The blocks consisting of 5 participants were created prior to the commencement of the study and provided to the research pharmacist. Both study participants and study staff involved with study assessments and data analysis were blind to the assignment. Only the research pharmacist who provided the study drink was aware of the assignment.

#### Dietary supplement and compliance.

The dietary supplement was developed by the Louisiana State University Agriculture center. The exact ingredients of the supplement cannot be disclosed due to legal constraints imposed by the proprietary formulation but includes at least two components chosen from the following six foods: 1) Collagen peptides of less than 3500 Daltons and preferably less than 2,000 Daltons derived from animals like cows, or other glycine-rich peptides; 2) Theanine, a component of decaffeinated green tea; 3) lactucopicrin, deoxylactucopicrin, or another lactucopicrin derivative found in chicory; 4) Hyaluronic acid, a component of cartilage; 5) epigallocatechin gallate, a component of decaffeinated green tea; and 6) quinic acid found in chicory. Some of these components are considered to promote sleep and thereby likely to improve quality of life in individuals with chronic insomnia [7–10]. The supplement was provided as a liquid (1.79 ounce/dose). A similar tasting visually indistinguishable drink was provided as a placebo control. Compliance was monitored by diary and accounting for empty bottles at the end of the 7-day intervention period.

#### Quantification and statistical analysis.

**Statistical power:** The overarching goal of the pilot study was to collect preliminary data to determine the effect of the supplement on the quality of life in individuals with insomnia to form the basis for powering a definitive clinical trial. For the pilot study, the Chubon LSS score is the outcome of primary interest, with all other outcomes derived from questionnaire, sleep study and accelerometry considered secondary/exploratory and were analyzed without a prespecified hierarchy for hypothesis generation. A successful pilot study would demonstrate effectiveness of the supplement in improving quality of life as determined by increase in the Chubon LSS score, compared to changes observed for placebo. The crossover study design with 10 participants allowed detection of a 7 ± 6 change from baseline between treatment groups in Chubon LSS score with a power of more than 80%. Further, with 10 participants, the study allowed detection of group differences in changes from baseline between placebo and supplement treatment by 15% for sleep efficiency, 35 min for WASO, and a difference in PSQI score of 4 with >84% power. Notably, these within and between group differences in habitual sleep patterns and self-perceived sleep quality would be considered clinically meaningful in participants with insomnia. To date, no controlled trials of this supplement have been conducted, therefore the pilot also aimed to determine if the supplement was able to alter quality of life and/or sleep. While the sample size of the pilot study allows detection of minimal relevant changes in Chubon LSS score, data related to expected variability in primary outcomes in the targeted study population are not available. Therefore, the pilot study provides essential data related to expected variability in quality of life and sleep metrics in participants with insomnia to calculate sample size for a future adequately powered clinical trial that would be able to account for the confounding variables, and to ensure that the findings can be generalized to a larger diverse population.

**Data collection and statistical analysis:** The statistical analysis plan was developed considering the nested crossover study design which included independent baseline and follow-up for each study period. Of the 10 participants randomized, one participant dropped out of the study after completion of the 1^st^ study period and analysis was conducted using a mixed effects linear model utilizing all available data, under the assumption of missing at random, which allows for the inclusion of the maximal amount of information collected from the participant. Importantly, the analysis plan did not interpolate for missing data. Fixed model covariates include crossover order (placebo first/ supplement first), study period (1^st^ or 2^nd^), timepoint (baseline and follow-up), treatment (placebo/ supplement), and timepoint with treatment interaction. Data nested within participant was considered as a random effect for the analysis to adjust for the correlation of data within participants. The main focus of the study is to compare the outcomes between the two groups, but since the impact of the supplement on the individual outcomes is unknown, it was important to also examine the within group changes between baseline and follow-up. Therefore, for each outcome, contrasts were applied within the mixed-model approach specifically to test within group difference from baseline to follow-up as well as between group differences (placebo Versus supplement). The primary analytical outcome was timepoint* treatment interaction and within group changes were considered exploratory analytical outcome. For habitual sleep patterns, data averages derived from the 7-day accelerometry during the intervention period were used with the same adjustment covariates without the time by treatment interaction due to the nature of the outcome. Based on results from previous studies, for all the variables reported in the manuscript, the carryover effects were assumed to be negligible after the washout period and no formal analysis to test for carryover effects were undertaken. Study period and crossover order were not significant for any of the variables (all P > 0.10), hence not reported individually below in the results section. The study data was collected and managed using REDCap electronic data tools hosted at PBRC [21, 22]. Analysis was performed using JMP software (SAS Institute Inc, Pro version16.0.0) with p < 0.05 being considered significant.

## Results

### Participants

Characteristics of the study participants are presented in Table 1. Ten participants (9 women; age: 59 ± 6 y; BMI: 30 ± 10 kgm^2^) were enrolled, and one participant was lost to follow-up after the 1^st^ study period (Fig 1). All women were post-menopausal. All participants reported having trouble sleeping more than 3 nights/week for the past 6 months including having difficulty falling asleep, staying asleep, or early awakenings. However, objectively determined habitual sleep patterns from 7-day accelerometry during screening showed a sleep latency of 5 ± 4 min (range 0–13 min) and WASO of 75 ± 33 min (range 35–113 min). The overall ISI score was 21 ± 4 (range 16–27) with moderately severe clinical insomnia in all participants. The participants had no sleep apnea as determined by oxygen desaturation index of 3 ± 1.6 events/h of sleep. No adverse events were reported during the study.

**Table 1:** Baseline characteristics of the study participants.

*Anthropometry*	
Age (years)	59 ± 6
BMI (kg/m^2^)	30 ± 10
Weight (kg)	82 ± 27
*Blood chemistry*	
Fasting glucose (mg/dL)	98 ± 14
Triglycerides (mg/dL)	79 ± 41
Total cholesterol (mg/dL)	206 ± 56
HDL cholesterol (mg/dL)	71 ± 23
LDL cholesterol (mg/dL)	119 ± 40
Creatinine (mg/dL)	0.76 ± 0.17
ALT (IU/L)	21 ± 9
ALK (IU/L)	59 ± 18
*Sleep*	
Insomnia severity index	21 ± 4
Sleep latency (min)	5 ± 4.1
Wake after sleep onset (min)	75 ± 33
Oxygen desaturation index (events/h)	3 ± 1.6
Sleep Efficiency (%)	85 ± 5.7

Data presented as mean (±) SD. N = 10. Abbreviations: *BMI*, body mass index; *LDL*, low-density lipoprotein; *HDL*, high-density lipoprotein; *ALT*, Alanine transaminase; *ALK*, Alkaline phosphatase.

#### Validated questionnaires.

In spite of clinically significant insomnia, seven out of ten participants had Chubon LSS score of >100 at baseline (period 1, visit 1) which corresponds to very good quality of life, while two participants had a score of >95–100, and only one participant had a score of <90. Group difference between placebo and supplement treatment trended to be different for the change in Chubon LSS score (Δ −1.4; CI: −2.8, 0.1; p = 0.06). Within group differences, compared to treatment baseline, were not observed after consumption of either placebo or supplement (Table 2). Treatment baseline values were not different between the two groups (Δ −2.9; CI: −7.0, 1.1; p = 0.15).

**Table 2:** Effects of supplement and placebo on self-reported indices of quality of life, daytime sleepiness, sleep, and fatigue.

	Placebo				Supplement				p-value (group difference)
	Baseline	Follow-up	Δ	p-value	Baseline	Follow-up	Δ	p-value	
Chubon LSS score	100.9 (95.6, 106.2)	102.9 (97.6,108.2)	2 (−1.9, 5.9)	0.30	103.9 (98.5,109.2)	100.4 (95, 105.8)	−3.4 (−7.6, 0.7)	0.1	0.06
ESS score	8.8 (6.2,11.4)	6.9 (4.3,9.5)	−1.9 (−3.4, −0.4)	**0.02**	8.4 (5.8,11.1)	9.9 (7.3, 12.5)	1.4 (−0.2, 3.0)	0.07	**<0.01**
PSQI score	10.5 (8.4,12.6)	8.3 (6.2,10.4)	−2.2 (−3.9, −0.5)	**0.02**	10.8 (8.6,13.0)	9.1 (6.9,11.3)	−1.7 (−3.7, 0.3)	0.09	0.69
MFI-general fatigue	14.7 (12.5, 17.0)	13.4 (11.2,15.6)	−1.3 (−2.5, −0.1)	**0.03**	15.1 (12.8, 17.3)	14.0 (11.7, 16.2)	−1.1 (−2.3, 0.1)	0.07	0.79
MFI-physical fatigue	10.3 (7.5,13.1)	11.3 (8.5,14.1)	1.00 (−0.7, 2.7)	0.22	11.1 (8.3, 14)	9.5 (6.6, 12.3)	−1.7 (−3.4, 0.1)	0.06	**0.03**
MFI-reduced activity	11.1 (8.4,13.8)	10.3 (7.6, 13)	−0.8 (−2.2, 0.6)	0.26	11.1 (8.3, 13.8)	10.8 (8.1, 13.6)	−0.2 (−1.7, 1.3)	0.77	0.57
MFI-reduced motivation	12.1 (10.5,13,7)	12.3 (10.7,13.9)	0.2 (−1.2, 1.6)	0.78	12.9 (11.1,14.6)	12.1 (10.4,13.7)	−0.8 (−2.4, 0.8)	0.32	0.35
MFI-mental fatigue	12.5 (10.3,14.7)	11.7 (9.5,13.9)	−0.8 (−2.3, 0.7)	0.27	11.7 (9.5, 13.9)	12.4 (10.1,14.6)	0.7 (−0.9, 2.2)	0.38	0.17

Participant scores for validated questionnaires are reported. Data presented are least squares mean, confidence intervals, and P values calculated from linear mixed model analysis. Significant p-values are in bold. Abbreviations: LSS: lifestyle situation survey; ESS: Epworth sleepiness scale; PSQI: Pittsburgh sleep quality index; MFI: multidimensional fatigue index. Supplement N = 9, Placebo N = 10.

At baseline (period 1, visit 1), excessive daytime sleepiness (ESS score of >10) was observed in 3 participants (score range 3–12). Group difference between placebo and supplement treatment was apparent for change in ESS score (∆ 0.8, CI: 0.3, 1.4; p = 0.005). Compared to treatment baseline, ESS score exhibited a trend toward increase with supplement intake (∆1.4 ± 0.8, p = 0.07) and decreased with placebo intake (∆−1.9 ± 0.73, p = 0.02). The baseline ESS score was not different for the two treatment groups (Δ 0.4; CI: −1.2, 1.9; p = 0.65).

All participants had PSQI scores indicative of poor sleep quality at baseline (period 1, visit 1; PSQI score range 5−16). No significant group difference was observed for change in PSQI score (Δ 0.1; CI: −0.5, 0.8; p = 0.69). Supplement intake was associated with a tendency to decrease PSQI score (∆ −1.7 ± 1, p = 0.09), compared to treatment baseline. However, a significant decrease in PSQI score was observed with placebo intake (∆ −2.6 ± 0.8, p = 0.02), compared to treatment baseline. Baseline PSQI score were comparable for placebo and supplement treatment (Δ −0.3; CI: −2.2, 1.6; p = 0.75).

For MFI-general fatigue, the baseline (period 1, visit 1) participant scores ranged from 9 to 19. Group difference in response to placebo and supplement were not observed for change in MFI-general fatigue score (Δ 0.05; CI: −0.4, 0.5; p = 0.79). Compared to treatment baseline, a tendency to decrease MFI-general fatigue was observed with supplement consumption (∆ −1.1 ± 0.6, p = 0.07) and a decrease in MFI-general fatigue was observed with placebo treatment (∆ −1.3 ± 0.6, p = 0.03). Treatment baseline measures of MFI-general fatigue were not different for the placebo and supplement groups (Δ −0.35; CI: −1.6, 0.92; p = 0.57). For MFI-physical fatigue, the baseline (period 1, visit 1) participant score range was 5–19, and a significant group difference between placebo and supplement groups for the change in MFI-physical fatigue score was observed (Δ −0.67; CI: −1.3, −0.1; p = 0.03). Further, compared to treatment baseline, a tendency to reduce MFI-physical fatigue score was observed with supplement consumption (∆ −1.7 ± 0.8, p = 0.06) while the scores remained unchanged with placebo (∆1 ± 0.8, p = 0.22). Treatment baseline MFI-physical fatigue score was not different for placebo and supplement (Δ −0.83; CI: −2.5, 0.9; p = 0.33). No within and between group change in other domains of fatigue was observed with either supplement or placebo consumption.

#### Sleep architecture and habitual sleep.

All participants showed poor sleep efficiency (75 ± 12%), delayed REM latency (129 ± 66 min), high WASO (82 ± 65 min), and reduced NREM3 sleep (10 ± 9%) at baseline (period 1, visit 1). The mean sleep latency was 43 ± 26 min at baseline (period 1, visit 1) in our study participants (range 8.5–81.5 min). No between group change in any sleep parameter including sleep efficiency (Δ −0.37; CI: −3.0, 2.3; p = 0.77), sleep latency (Δ −0.10; CI: −7.2, 7.0; p = 0.98), REM latency (Δ −0.67; CI: −16.9, 15.6; p = 0.93), WASO (Δ 0.25; CI: −11.3, 11.8; p = 0.97), arousal index (Δ 0.99; CI:-2.9, 4.8; p = 0.60), and periodic limb movement index (Δ 1.74; CI: −4.5, 7.9; p = 0.57) was observed for supplement or placebo treatment (Table 3). No significant within group changes from treatment baseline were observed for either placebo or supplement group. Treatment baseline sleep characteristics as determined by polysomnography were similar for both placebo and supplement groups.

**Table 3:** Effect of supplement and placebo on sleep architecture.

Characteristics	Placebo				Supplement				p-value (group difference)
	Baseline	Follow-up	Δ	p-value	Baseline	Follow-up	Δ	p-value	
Sleep Efficiency (%)	80 (71, 88)	81 (72, 89)	−1 (−9, 6)	0.73	80 (71, 88)	79 (71, 88)	0.3 (−7, 8)	0.94	0.77
Sleep latency (min)	32 (16, 48)	28 (13, 44)	4 (−16, 23)	0.71	28 (11, 44)	24 (7, 41)	4 (−17, 25)	0.69	0.98
REM latency (min)	120 (68, 173)	108 (55,161)	13 (−32, 57)	0.56	117 (62, 171)	101 (47, 156)	15 (−32, 62)	0.51	0.93
Wake after sleep onset (min)	71 (28,115)	72 (28, 116)	−1 (−33, 31)	0.96	77 (32, 121)	79 (34, 123)	−2 (−35, 32)	0.91	0.97
Stage NREM1 (%)	10 (6, 13)	12 (9, 16)	−2 (−7, 2)	0.32	9 (5, 13)	9 (5, 13)	−0.2 (−5, 5)	0.92	0.54
Stage NREM2 (%)	61 (56, 66)	58 (53, 63)	3 (−3, 9)	0.29	57 (51, 62)	59 (53, 64)	−2 (−8, 4.3)	0.54	0.24
Stage NREM3 (%)	8 (4, 13)	8 (3, 13)	0.4 (−2, 3)	0.78	10 (6, 15)	9 (4, 14)	2 (−1, 5)	0.19	0.45
Stage REM (%)	21 (16, 26)	22 (17, 27)	−1 (−7, 5)	0.70	24 (18, 29)	24 (18, 29)	−0 (−6, 6)	0.99	0.80
Arousal index (#/h)	14 (6, 21)	15 (7, 22)	−1 (−11, 10)	0.88	15 (7, 23)	20 (12, 28)	−5 (−16, 6)	0.39	0.60
PLM index (#/ h)	25 (0.4, 49)	25 (1, 50)	−1 (−18, 17)	0.95	33 (8, 58)	41 (16, 65)	−7 (−25, 11)	0.40	0.57

Polysomnography data from in-lab sleep study comparing the two interventional conditions of the study. Data presented are least squares mean, confidence intervals, and P values calculated from linear mixed model analysis. Significant p values are in bold. REM: rapid eye movements, NREM: non-rapid eye movement. PLM: periodic limb movement. Supplement N = 9, Placebo N = 10.

Habitual sleep patterns as determined by 7-day accelerometry showed a tendency to increase nighttime sleep duration (448 ± 14 Vs 437 ± 14 min, p = 0.07, placebo vs supplement, respectively) in the placebo group while the time in bed at night remained unchanged (521 ± 18 Vs 522 ± 18 min, p = 0.97, Table 4). This was accompanied by no change in WASO (68 ± 14 Vs 79 ± 14 min, p = 0.32) or sleep fragmentation index in the placebo group (23 ± 3 Vs. 29 ± 3.1, p = 0.12). Interestingly, while not statistically different, an increase in 24 h total time in bed (nighttime plus daytime naps) was noted during supplement treatment (531 ± 21 Vs. 541 ± 21 min, p = 0.48) likely resulting from an increase in daytime naps when consuming the supplement, as reported by participants.

**Table 4:** Effect of the supplement and placebo on habitual sleep.

Characteristic	Placebo	Supplement	Δ	p-value
Sleep latency (min)	6 (1, 10)	6 (2, 11)	−0.8 (−8, 6)	0.80
Sleep efficiency (%)	86 (81, 92)	84 (79, 90)	2 (−2, 6)	0.31
Total time in bed at night (min)	521 (482, 561)	522 (481, 562)	−0.5 (−31, 30)	0.97
Total sleep time at night (min)	448 (415, 480)	437 (404, 470)	11 (−1, 23)	0.07
Wake after sleep onset (min)	68 (38, 98)	79 (48,110)	−11 (−36, 13)	0.32
Sleep fragmentation index (%)	23 (16, 30)	29 (22, 36)	−6 (−13, 2)	0.11
Number of awakenings during sleep (per h)	16 (11, 21)	16 (11, 21)	0.13 (−5, 5)	0.95
Average awakening length (min)	5 (4, 5)	5 (4, 6)	−0.4 (−2, 1)	0.53
Total 24h time in bed (including naps, min)	531 (484, 577)	541 (494, 589)	−11 (−46, 24)	0.48
Total 24h sleep time (including naps, min)	462 (424, 500)	456 (418, 495)	5 (−16, 27)	0.56

Daily sleep patterns were determined by 7-day accelerometry during the days the participant took supplement or placebo. Data presented are least squares mean, confidence intervals, and P values from linear mixed model analysis. Supplement N = 9, Placebo N = 10.

## Discussion

Our exploratory pilot study showed that the daily consumption of the herbal supplement for 7 days, compared to placebo consumption, did not improve self-perceived quality of life, daytime sleepiness, fatigue, or sleep quality in participants with insomnia. Further, the supplement did not alter objectively measured sleep architecture, sleep quality, or habitual sleep patterns, compared to placebo, in our study participants. These findings are contradictory to the anecdotal evidence and some clinical studies, which suggest that supplements containing green tea and chicory extracts improve sleep and reduce daytime insomnia symptoms to improve overall quality of life.

Insomnia is a complex and heterogenous disorder with potentially different pathologies associated with different sleep problems. Indeed, different drugs are prescribed for sleep problems resulting from issues related to falling asleep versus maintaining sleep or early awakenings [23]. While our study allowed inclusion of participants with any insomnia symptoms, none of the participants had issues with falling asleep. It is likely that the supplement may have sleep promoting properties that may be more helpful for individuals with sleep problems related to sleep latency rather than sleep maintenance or early awakenings. Nevertheless, our findings are partly consistent with other studies containing epigallocatechin gallate which is a derivative of decaffeinated green tea [24]. In a similar randomized, placebo-controlled clinical trial targeting participants with sleep disturbances there was no significance in the effects of active supplement on sleep latency and time in REM sleep with results suggesting a placebo effect [24]. However, unlike our study, the supplement showed significant improvement in self-report sleep quality, attention, and symptoms of insomnia. The comparative but varying results between studies could be due to the characteristic of study participants (age, sleep issue, sex), number of subjects participating, and the intervention duration.

Our study shows improvement in some self-reported indices with placebo treatment in the study participants but not in any objective measures of sleep. The placebo effect in clinical trials remains largely unexplained and can be caused by several mechanisms including psychological factors such as belief and expectations [25]. Interestingly, placebo effects have been shown to be most effective on symptoms modulated by the brain, like sleep and pain. Of note, the placebo effect is quite common in studies aimed at improving sleep in patients with insomnia [26]. In a meta-analysis including thirteen independent studies, a reliable placebo effect was observed which led to improvement of subjective sleep measures including perceived improvement in sleep latency, total sleep time and sleep quality, compared to no treatment. At the same time, the placebo effect was not apparent in objective measures of sleep. The discordance between changes in subjective and objective measures of sleep with placebo treatment may be related to dependence of the subjective measures on perceptions which can be readily influenced by expectations and beliefs. In contrast, the objective sleep measures determined by accelerometry and sleep study are not influenced by our attitudes. The importance of sleep perception is highlighted by an interesting study which showed that changing sleep perception alters cognitive performance [27]. However, the improvements in subjective measures of sleep, while statistically significant, were not clinically meaningful. Our study highlights the importance of 1) having a placebo control even in pilot studies; and 2) assessing both objective and subjective measures of sleep.

Our pilot study has several strengths including measures of different aspects pertaining to quality of life that are relevant to patients with insomnia along with objective and subjective sleep-related measures. The crossover study design allows for a robust determination of impact of supplement or placebo irrespective of the underlying pathology contributing to insomnia symptoms. Furthermore, the crossover design also minimizes concerns related to confounds arising from baseline difference in factors such as body weight, body fat content, nutrition, or lifestyle. However, inherent concerns related to limitation of a crossover study to eliminate the residual effects from prior intervention treatment remain. Of note, while the statistical adjustment for the crossover order addresses systematic differences between crossover order, it does not constitute formal test of carryover effects. Other limitations include those related to the small sample size of the pilot study that reduce the generalizability of the findings and does not allow examination of the confounding variables. The study only enrolled 10 participants with an imbalance in the sex (mostly females). It is likely that the supplement may have shown beneficial effects in a larger diverse population including younger individuals (study only included individuals with >50 years of age), males, and different ethnicities. Further, our study had a short intervention duration and did not evaluate body composition or habitual diet and lifestyle, which may have confounded our findings. Sleep and diet are closely linked with certain habits such as alcohol intake, caffeine consumption, meal timing, etc., which may alter sleep. An important limitation was the presence of other sleep-related disorders which may have contributed to continued poor sleep such as presence of restless leg syndrome (RLS) and obstructive sleep apnea. None of the participants reported prior RLS diagnosis, but our sleep study results suggest that RLS may have been present in all our study participants. Also, even with an oxygen desaturation index within normal limits (<5 events/h of sleep), one of the participants demonstrated the presence of position-dependent obstructive sleep apnea. The presence of sleep apnea became apparent only during the second study period when the participant was forced to sleep on her back due to a back injury. Lastly, despite insomnia, the participants reported a good quality of life which prevents overall improvements in quality of life from manifesting.

To summarize, our pilot study showed that the daily consumption of the herbal supplement containing chicory, decaffeinated green tea, and epigallocatechin gallate that was provided to our participants for 7 days did not change any aspect of quality of life, sleep duration, or sleep quality in participants with insomnia characterized by increased WASO but not longer sleep latency. Future studies aiming to explore the beneficial effect of this supplement should consider a longer intervention period and target a more diverse group of participants with equal inclusion of sexes, different ethnic backgrounds, and ensuring absence of sleep disorders other than insomnia. Notably, participants with insomnia symptoms related to difficulties in initiating sleep and/or those with poor quality of life should be included to be able to explore the sleep promoting effects of the supplement.

## Supporting information

S1 Table Participant eligibility criteria. Participants were recruited based on the following inclusion and exclusion criteria to participate in the study.
(DOCX)

S1 Protocol IRB approved study protocol. (PDF)

S1 Checklist CONSORT checklist: Pilot and feasibility trials checklist. (DOC)
