# Supplementary material for: Effect of an herbal supplement on quality of life in participants with insomnia: A randomized placebo controlled cross-over pilot trial
Source: PLoS One. 2026 May 20;21(5):e0350039. doi: 10.1371/journal.pone.0350039 (PMC13189356; doi:10.1371/journal.pone.0350039)
Supplement: S1 Table — Participants were recruited based on the following inclusion and exclusion criteria to participate in the study. (DOCX) [file pone.0350039.s001.docx]

**S1 Table: Participant eligibility criteria.** Participants were recruited based on the following inclusion and exclusion criteria to participate in the study.

| **Inclusion Criteria** | |
| --- | --- |
| 1 | Healthy male or female subjects. |
| 2 | 50 years or older. |
| 3 | Usual bedtime is between 9 pm and midnight. |
| 4 | Chronic difficulty sleeping for on average for more than 3 nights per week for at least 6 months. |
| 5 | Insomnia Severity Index score greater than or equal to 10. |
| 6 | A minimum of 30 minutes of either sleep-onset latency or wake after sleep onset (WASO) as determined by 7-day accelerometry. |
| 7 | A complaint of difficulty initiating sleep, difficulty maintain sleep or waking up too early or sleep that is chronically non-restorative or poor in quality. |
| 8 | The sleep difficulties occur despite adequate opportunity and circumstances for sleep. |
| 9 | Participant reports at least one of the following forms of daytime impairment related to the nighttime sleep difficult.  a. Fatigue or malaise.  b. Attention, concentration, or memory impairment.  c. Social or vocational dysfunction or poor school performance.  d. Mood disturbance or irritability.  e. Daytime sleepiness.  f. Motivation, energy, or initiative reduction.  g. Proneness for errors or accidents at work or while driving.  h. Tension, headaches, or GI symptoms in response to sleep loss.  i. Concerns or worries about sleep. |
| **Exclusion Criteria** | |
| 1 | Have diabetes diagnosis. |
| 2 | Are taking prescription sedating or hypnotic medications. |
| 3 | Taking any chronic medication for which, the dose has not been stable dose for 1 month or longer. |
| 4 | Have sleep apnea diagnosis or an oxygen desaturation index of more than >5 events/h as determined by overnight oximetry. |
| 5 | Adults are unable to consent. |
| 6 | Pregnant women. |
| 7 | Prisoners. |
