# Supplementary material for: Effect of an herbal supplement on quality of life in participants with insomnia: A randomized placebo controlled cross-over pilot trial
Source: PLoS One. 2026 May 20;21(5):e0350039. doi: 10.1371/journal.pone.0350039 (PMC13189356; doi:10.1371/journal.pone.0350039)
Supplement: S1 Protocol — (PDF) [file pone.0350039.s002.pdf]

Protocol Title: A Cross-over Pilot Trial to Determine the Effect of an LSU Patented Dietary Herbal Supplement on Quality of Life

PI Name: Frank Greenway, MD

Sub-Investigator's Name(s): Prachi Singh, PhD

Protocol Version Date: December 16, 2021

IRB Review History
Modification 3

Objectives
Specific Aim 1: Compare the quality of life using the validated Chubon Quality of Life Rating scale after taking the supplement or the placebo at baseline followed by a 2-week washout, then 1 week taking the supplement or placebo that was not taken in the first period.

Specific Aim 2: Compare the safety of the supplement by evaluating questionnaires, chemistry panel, CBC, Janus 3, adverse events and polysomnography at baseline and after 1 week of nightly administration with the supplement or the placebo before and after a 2-week washout.

Hypothesis: The supplement will improve the quality of life compared to the placebo condition

Background
Jack Losso and Jose Andino at Louisiana State University Agricultural Center developed a novel dietary herbal supplement consisting of at least two components chosen from the following six foods. 1. Collagen peptides of less than 3500 Daltons and preferably less than 2,000 Daltons derived from animals like cows, or other glycine-rich peptides. 2. Theanine, a component of decaffeinated green tea. 3. lactucopicrin, deoxylactucopicrin or another lactucopicrin derivative found in chicory. 4. Hyaluronic acid, a component of cartilage. 5. epigallocatechin gallate, a component decaffeinated green tea. and 6. quinic acid found in chicory. This dietary supplement contains chicory and decaffeinated green tea containing epigallocatechin gallate. These ingredients are thought to decrease Janus kinase 3 (JAK-3) and people who have tried the supplement claim that it made them feel better, made them feel more alert and improved their quality of life.

This trial is designed to measure the quality of life in response to taking the novel supplement. People over 50 years of age with difficulty sleeping frequently feel tired and lack in energy which degrades their quality of life. We will test the effect of the dietary herbal supplement on quality of life using a validated questionnaire in humans with difficulty sleeping over 50 years of age compared to a placebo in a crossover trial

comparing the supplement to a placebo after 1 week of administration separated by a 2-week washout period.
The dietary herbal supplement comes in a liquid that is dissolved in flavored water and administered orally in the evening in a 1.79 ounce portion an hour or two before bedtime. Uncontrolled pilot data was generated in 12 people suffering from trouble sleeping who took the dietary herbal supplement for 1 week. They awoke refreshed, felt more alert and felt that the quality of their lives had improved on the supplement. This study will evaluate quality of life in a controlled crossover trial using a validated quality of life measure (1). Since the participants in the pilot clinical trial all had trouble sleeping, we want to understand the safety of the supplement in people with sleep problems, and we also want to understand as much as possible the nature of the sleep difficulty and how the sleep difficulty is related to the stated improvement in quality of life. The polysomnography is the test that allows the determination of the characteristics of the sleep difficulty and the questionnaires are also helpful in determining in what way the quality of life is improved, if the controlled trial confirms the findings of the pilot uncontrolled trial.

Inclusion and Exclusion Criteria
Subjects will be recruited through the Pennington Recruiting Department

Subjects who meet the following criteria will be included in the study.
1. Healthy male or female subjects
2. 50 years of age or older
3. Willing to have blood stored for future research.
4. Usual bedtime between 9 pm and mid-night
5. Chronic difficulty sleeping (trouble sleeping on average for more than 3 nights per week, meeting the International Classification of Sleep Difficulty (ICSD-2*) for at least 6 months.
6. Insomnia Severity Index score greater than or equal to 10 and a minimum of 30 minutes of either sleep-onset latency (SL) or wake after sleep onset (WASO).
7. *ICSD-2 general criteria for sleep difficulty:
   A. A complaint of difficulty initiating sleep, difficulty maintaining sleep, or waking up too early, or sleep that is chronically non-restorative or poor in quality.
   B. The above sleep difficulty occurs despite adequate opportunity and circumstances for sleep.
   C. At least one of the following forms of daytime impairment related to the nighttime sleep difficulty is reported by the patient:
      a. Fatigue or malaise.
      b. Attention, concentration, or memory impairment.
      c. Social or vocational dysfunction or poor school performance.
      d. Mood disturbance or irritability.
      e. Daytime sleepiness.
      f. Motivation, energy, or initiative reduction.
      g. Proneness for errors or accidents at work or while driving.
      h. Tension, headaches, or gastrointestinal symptoms in response to sleep loss.
      i. Concerns or worries about sleep.

Subjects that meet the following criteria will be excluded from the study

1. Have diabetes.
2. Are taking sedating or hypnotic medications.
3. Taking any chronic medication for which the dose has not been stable dose for 1 month or longer.
4. Have a sleep disorder other than difficulty sleeping such as sleep apnea
5. Adults unable to consent
6. Individuals who are not yet adults (infants, children, teenagers)
7. Pregnant women
8. Prisoners

Number of Subjects
Ten subjects will be enrolled in this cross-over study consisting of a screening visit and four study visits

Recruitment Methods
The subjects will be recruited through the Pennington Advertising and Recruiting Departments and connections with Dr. Schwab who is a sleep specialist.

We anticipate a successful recruitment from a past history of referrals from Dr. Schwab

The advertisements will be developed by the marketing department and submitted for IRB review.

Study Timelines
The duration of each subject's participation will be approximately one month. There will be a 1-week period of taking the supplement or the placebo, a 2-week washout and another 1-week period of consuming the placebo or supplement not taken in the first arm of the crossover trial

The grant was issued in February of 2020 and should be completed within a year. The Covid-19 problem has delayed the start of the study, but we hope to complete the study by the end of 2022.

We anticipate finishing the trial in 12 weeks, if we enroll a subject each week. The primary analysis should be completed in the following 2 weeks and the report submitted to the Lift Grant Administration 4 weeks from the study completion.

Study Endpoints
Primary Endpoint:

1. The Chubon Quality of Life Rating Scale. This scale has a test-retest stability of 0.91, Chronbach's alpha for variety of group ranges from 0.74 to 0.95 for internal consistency (2).

Secondary Safety Endpoints:

1. The Insomnia Severity Index questionnaire
2. The Epworth Sleepiness Scale questionnaire
3. The Pittsburgh Sleep Quality Index questionnaire

4. Janus-3 kinase
5. Polysomnography
6. 7-day accelerometry
7. Chemistry panel and CBC
8. Adverse events

Procedures Involved

Subjects will have a screening visit and home oximetry to rule out sleep apnea. Sleep apnea is characterized by episodes in which people stop breathing during sleep. During this cessation of breathing the oxygen in the blood decreases and the lowered oxygen awakens the person. A pulse oximeter is a device that measures the oxygen in the blood stream. If the person experiences drops in blood oxygen during the night, it would be consistent with the person having sleep apnea. If someone screened for the study has episodes of low blood oxygen as measured by the pulse oximeter, they will be referred to their primary physician or a sleep specialist to have a formal sleep study, using polysomnography. That test can not only diagnose sleep apnea, but also define whether there is need for treatment of sleep apnea. Sleep apnea is treated with Continuous Positive Airway Pressure (CPAP). Subjects meeting the inclusion and exclusion criteria will have questionnaires, blood testing and polysomnography and then start one week of a dietary herbal supplement or placebo. The testing done on test day one will be repeated after 1 week. After a 2-week washout period, the same procedures will be repeated at baseline that were performed on week-1. The supplement or placebo not given in the first testing period will be given in the 1-week testing period.

Schedule of Events

Week	Scr	Base	1	3	4
Consent	x				
Medical Questionnaire	x				
Vital signs	x	x	x	x	x
Height	x				
Weight	x	x	x	x	x
Polysomnography		x	x	x	x
Oximetry (R/O Sleep Apnea)	x				
Chubon Quality of Life Rating		x	x	x	x
Insomnia Severity Index	x	x	x	x	x
Epworth Sleepiness Scale		x	x	x	x
Pittsburgh Sleep Quality Index		x	x	x	x
Multidimensional fatigue Inventory		x	x	x	x
7-Day Actigraphy &Sleep Diary	X	x		x	
Actigraphy During Sleep Test		x	x	x	x
Chemistry Panel, CBC	x		x		x
Janus kinase 3,			x		x
Archive Serum and Buffy Coat			x		x
Adverse Events			x		x
Dispense/collect Test material		x	x	x	x

Screening visit: Subjects will come to the screening visit having fasted for 10 hours except for water. Subjects will give informed consent, have vital signs height and weight measured, have blood drawn for a chemistry panel, CBC, complete a health questionnaire and complete the validated questionnaire, the Insomnia Severity Index. Subjects will have overnight oximetry to screen for sleep apnea rather than true difficulty sleeping. Subjects will be asked to wear an actigraphy to measure motion/activity during the day and night for 7 days. Subjects will be asked to keep a sleep diary for 7 days and return the diary, along with the actigraphy to the study staff for review. Those passing the screening visit will return for study visit one.

Study visit 1: Subjects will come to the sleep lab at 7:00 pm after having eaten dinner at home for an overnight polysomnography test that includes oximetry and actigraphy. Subjects will be asked to sleep from 10:00 pm to 6:00 am the next day. The following morning subjects will complete 5 validated questionnaires: the Insomnia Severity Index, the Epworth Sleepiness Scale, the Pittsburgh Sleep Quality Index, the Chubon Quality of Life Rating Scale, and the Multi-dimensional Fatigue Inventory. Unless a specific cause like sleep apnea is identified as the reason for the fatigue symptoms, the subjects will be given a week's supply of the dietary supplement or placebo with instructions to take 2 ounces an hour before bedtime. The study order of the supplement or placebo will be assigned randomly. Subjects will be asked to use actigraphy and keep a sleep diary for the 7-day period until their next visit.

Study Visit 2: The subject will return in 1 week (±3 days) for a repeat polysomnography test, 1 hour before which the subject will take the assigned study supplement or placebo. Upon awakening the next morning, the subjects will have a blood drawn for a chemistry panel, a CBC and Janus kinase 3. Subjects will also have blood drawn to archive serum, buffy coat and EDTA plasma. On awakening the next morning they will be asked to complete the 5 validated questionnaires: Insomnia Severity Index, the Epworth Sleepiness Scale, the Pittsburgh Sleep Quality Index, the Chubon Quality of Life Rating Scale and the Multi-dimensional Fatigue Inventory.

After a **2-week washout period** subjects will return for visit 3.

Study visit 3: This visit occurs after a 2-week washout period, and is the same as visit 1, only the supplement or placebo will be the one the subject was not assigned at study visit 1 (dietary supplement or placebo).

Study visit 4: This visit is the same as visit 2, and will be the end of the trial.

Blood testing: There is the possibility of infection and/or pain and bruising at the vein on the arm where the needle is inserted. Aseptic (sterile) technique and trained personnel minimize these risks.

Questionnaires: There are no known risks to completing the questionnaires

Measuring height, weight, blood pressure and pulse rate has no known risks

Actigraphy is wearing a wrist watch-like device that measures motion. Actigraphy has no known risks

Sleep Diary is to keep track of your sleep habits and experiences. There are no known risks in keeping a sleep diary.

Oximetry is a device that is worn on the finger like a thimble and measures the oxygen in your blood using a light. There are no known risks to wearing an oximetry device

Dietary Supplement is a mixture of 3 foods, a component of chicory, decafinated green tea powder and another component of green tea. The supplement has no known risks.

> **Ingredients**: each bottle contains a mixture of 5 grams of collagen peptides, 5 grams of green tea extract, 1 gram of chicory extract, 7 grams of sweetener (half sugar and half erythritol), 0.2% potassium sorbate (as a preservative, 0.1% flavor, 60ml boiling water is added and colled to 185 degrees F. 53 ml or 1.79 ounces is hot packed in a seal proof bottle that can be stored at room temperature or under refrigeration in this ready to serve format.
> **Dose**: The totla volume of each bottle is 53mL or 1.79 ounces and each bottle is one dose
> **Lot number**: The lot number is 2008-1for the supplement and 2008-2 for the placebo. The supplement and placebo were prepared on August 17, 2020
> **Preparation**: The supplement and the placebo were prepared at the LSU Food Incubator.
> **Temperature**: The supplement and placebo have been stored at room temperature.
> **Shelf-life**: The shelf-life of the supplement is at least 2 years at room temperature. The product was hot packed and contains potassium sorbate which is a preservative for long-term storage. The expiration date for the supplement and placebo is August 17, 2022 and it is presently being stored in the Pennington pharmacy. The results of the stability study are attached.

Polysomnography (PSG) is an overnight video sleep study. PSG will include electroencephalography, electrooculography, electromyography, electrocardiography, pulse oximetry, nasal pressure transducer, oronasal thermocouple, calibrated respiratory inductance plethysmography. Sleep staging and events will be scored by registered PSG technologists according to established criteria.

Data and Specimen Banking
White blood cells called the buffy coat, serum from the blood and EDTA plasma will be stored for future tests related to the effect of the supplement on the white blood cells or serum. The white blood cells and serum will be frozen and used over a period of several months. Any extra material may be stored for future tests related to insomnia or the effect of the supplement on white blood cells or serum. The testing of the cells will be limited to the effect of the supplement on white blood cells and serum.

Power analysis.
No controlled trials of the supplement exist to power this study. This is a pilot study. if it is statistically insignificant, it will provide the basis for powering a definitive clinical trial.

Data and Specimen Management
The demographic data will be described using summary statistics. The comparisons of the supplement and placebo will be analyzed using paired t-testing. Data will be stored in the Pennington Data base that is controlled by the Research Computing Group and is password protected. Subjects in the trial will be given an A number which allows them to remain unknown to those analyzing the data. The data will be stored indefinitely as has all study data generated at Pennington. Buffy coats and serum will be stored at Pennington. The buffy coats and serum will be stored indefinitely and be used to analyze the effect of the supplement on white blood cell and serum related to sleep difficulty. Any data transfer will be done by the Pennington Research Computing Group.

Provisions to Monitor the Data to Ensure the Safety of Subjects
This is a minimal risk study, but the investigators will keep in close communication with the study coordinator to insure the study is executed properly and that the study participants are not having any concerning side effects.

Withdrawal of Subjects
Subjects will be withdrawn from the study should it be determined by the medical investigator that the potential benefits of the study are out-weighed by side effects. Subjects may also be withdrawn from the study if they are unwilling or unable to comply with the study requirements.

Risks to Subjects
The only foreseeable risks of this study are those associated with drawing blood. There is the possibility of infection and/or pain and bruising at the vein on the arm where the needle is inserted. Aseptic (sterile) technique and trained personnel minimize these risks. The other procedures in this study have no known risks

Potential Benefits to Subjects
The subjects in this study may experience an improvement in their quality of life in the week they take the supplement. If that does occur, the effect is likely to go away after they are off the supplement for a day or two.

Vulnerable Populations
This study excludes vulnerable populations

Multi-Site Research
This is a single site study.

Sharing of Results with Subjects
The study findings will be shared with the study participants. We are expecting to present the study at a medical meeting and will share the abstract or press release with the study participants. The tests done in the study will be shared with the subjects and with their medical practitioner, if the patient wants that sharing to take place.

Setting

The study will take place at the Pennington Center. Pennington coordinators and other health professionals are well-trained to conduct the procedures in this clinical trial.

Resources Available
We have done a study in which we recruited subjects with sleep difficulty. We have also developed a good relationship with Kyle Schwab, a sleep specialist. Between the patients with sleep problems wanting to enroll through information released by the Pennington Center and our relationship with the Sleep Center, we do not think that gathering study participants for the study will be difficult. Pennington has all the resources needed for this study.

Prior Approvals
We have received approval from the LIFT program with funding to complete the study.

Compensation
Participants will receive $200 for completing the study.

Confidentiality
The study data will be stored indefinitely at Pennington in the database and in the medical records area. The data will be controlled by the Research Computing Group, and they will be responsible for any data transfer.

Provisions to Protect the Privacy Interests of Subjects
The study subjects will be seen in private exam rooms to maintain their privacy. The sleep studies will be done in a separate room with privacy curtains to for changing into bed clothing and getting redressed.

Compensation for Research-Related Injury
No form of compensation for medical treatment or for other damages (i.e., lost wages, time lost from work, etc.) is available from the Pennington Biomedical Research Center. In the event of injury or medical illness resulting from the research procedures in which you participate, you will be referred to a treatment facility. Medical treatment may be provided at your expense or at the expense of your health care insurer (e.g., Medicare, Medicaid, Blue Cross-Blue Shield, Dental Insurer, etc.) which may or may not provide coverage. The Pennington Biomedical Research Center is a research facility and provides medical treatment only as part of research protocols. Should you require ongoing medical treatments, they must be provided by community physicians and hospitals

Economic Burden to Subjects
None

Consent Process
The consent process will start with the potential subjects reading the consent form and one of the study team then going over it with them to be sure they understand it and to be sure all their questions are answered. If subjects want to take the consent home and discuss it with their family or counselor, that will be allowed. The consent will be discussed in a private examination room and the subjects will be encouraged to ask

questions throughout the study, even after the consent is signed with the study team emphasizing that giving consent is a process that continues throughout the trial.

References

1. Chubon RA. Development of a quality-of-life rating scale for use in health-care evaluaton. Eval Health Prof. 1987;10(2):186-200.
2. Williams JI, Wood-Dauphinee SW. Quality of life and technology assessment: monograph of the council on health care technology. Institute of Medicine Council on Health Care Technology, Mosteller F and Falotico-Taylor J, Editors. Washington DC, National Academies Press (US) 1989.

Appendix A

Chubon Quality of Life Index

Appendix B

Insomnia Severity Index:
https://www.ons.org/sites/default/files/InsomniaSeverityIndex_ISI.pdf

Appendix C

Epworth Sleepiness Scale:
https://epworthsleepinessscale.com/about-the-ess/

Appendix D

Pittsburgh Sleep Quality Index
https://www.psychiatry.pitt.edu/sites/default/files/inline-files/PSQI%20Article.pdf

Appendix E

Multi-dimensional Fatigue Inventory
https://www.med.upenn.edu/cbti/assets/user-content/documents/Multidimensional%20Fatigue%20Inventory%20(MFI).pdf
